# Multiphysics Modeling Framework for Soft PVC Gel Sensors with Experimental Comparisons

**DOI:** 10.3390/polym15040864

**Published:** 2023-02-09

**Authors:** Justin Neubauer, Kwang J. Kim

**Affiliations:** Department of Mechanical Engineering, University of Nevada, Las Vegas, NV 89154, USA

**Keywords:** PVC gels, electroactive polymers, soft sensors, smart materials

## Abstract

Polyvinyl chloride (PVC) gels have recently been found to exhibit mechanoelectrical transduction or sensing capabilities under compressive loading applications. This phenomenon is not wholly understood but has been characterized as an adsorption-like phenomena under varying amounts and types of plasticizers. A different polymer lattice structure has also been tested, thermoplastic polyurethane, which showed similar sensing characteristics. This study examines mechanical and electrical properties of these gel sensors and proposes a mathematical framework of the underlying mechanisms of mechanoelectrical transduction. COMSOL Multiphysics is used to show solid mechanics characteristics, electrostatic properties, and transport of interstitial plasticizer under compressive loading applications. The solid mechanics takes a continuum mechanics approach and includes a highly compressive Storakers material model for compressive loading applications. The electrostatics and transport properties include charge conservation and a Langmuir adsorption migration model with variable diffusion properties based on plasticizer properties. Results show both plasticizer concentration gradient as well as expected voltage response under varying amounts and types of plasticizers. Experimental work is also completed to show agreeance with the modeling results.

## 1. Introduction

Polyvinyl chloride (PVC) gels have been studied as electroactive polymer smart materials since the early 2000s. These materials are heavily researched as compliant actuators, which have many applications in wearable and biomechanical applications [1,2,3]. Fundamental research on the underlying mechanism of electromechanical transduction is also well established [4,5,6]. The underlying mechanisms of electromechanical transduction, or actuation properties, is as follows: an applied electric field causes migration of free interstitial plasticizer towards the anode, which causes a plasticizer-rich layer with increased space charge density. This plasticizer-rich layer causes a significant decrease in material modulus and actuation results from a combination of electrostatic pressure and Maxwell stresses. Mathematical modeling of this phenomena is presented using finite-element tools such as COMSOL Multiphysics [7]. This finite-element tool has advantages in these applications due to the ability to seamlessly mesh multiple physics modules such as the solid mechanics, electrostatics, and transport properties used in this study.

The sensing properties of these materials were recently investigated and found to be adsorptive-like in nature [8,9,10]. The fundamental studies investigating the underlying mechanisms of mechanoelectrical transduction include compressive loading applications. The study investigates the steady-state response of these gels under incrementally increasing compressive loading applications. The steady-state response showed little dependence on thickness and applied compressive strain rate, but a high dependence on cross-sectional area and plasticizer amount in the gel sample. Combining the low dependence on thickness and high dependence on cross-sectional area, the underlying mechanism of mechanoelectrical transduction was deemed to be a surface-dominated property.

In another study, multiple plasticizer types were investigated for mechanoelectrical transduction properties in gel sensors. These results were fit using a Langmuir adsorption isotherm, which is a common adsorption model used to model a wide range of adsorptive phenomena [9]. This adsorptive model shows good agreement with experimental results and was also nondimensionalized to show goodness of fit. The classic S shape and halfway point was observed in PVC gel samples across all plasticizers used in the study. Thermoplastic polyurethane (TPU) was also used to create gel sensors and showed similar mechanoelectrical characteristics to PVC gel sensors with analogous levels of plasticizer when plotting response against the applied strain of the gel sensors. This study showed the nonuniqueness of PVC in these gel sensors. This also implies the limited role of the polymer lattice in the underlying mechanoelectrical transduction properties of these gel sensors other than altering the material modulus. This study showed that the Langmuir adsorption model shows good agreement with these gel sensors, regardless of the amount or type of plasticizer, as well as in differing polymer lattice structures. The nomenclature for the proposed mathematical model is given in Table 1.

This study aims to further investigate mechanoelectrical transduction and material properties of these gel sensors under compressive loading. The fundamental studies of these materials include both steady-state and transient characteristics due to observed viscoelastic properties. The steady-state model used is a hyperelastic Storakers material model that is commonly used for porous and lattice structures with high compressibility in compression loading applications, such as foams [11,12]. This approach is used due to the highly compressive and nonlinear nature of the compressive modulus. Polymers often have nonlinear strain energy and stress–strain relationships due to the morphology of the polymerized lattice structure [13,14]. The continuum mechanics approach of the Storakers hyperelastic model stems from the well-established Ogden material model, which is commonly used in noncompressible applications. The stored energy, W, is represented as a function of the principal stretches ($\lambda_{1}$, $\lambda_{2}$, and $\lambda_{3}$).

(1) $$W=\overset{\;}{\underset{n}{\sum }}\frac{{\tilde{\mu }}_{n}}{\alpha_{n}}\left(\lambda_{1}^{\alpha_{n}}{+\lambda }_{2}^{\alpha_{n}}{+\lambda }_{3}^{\alpha_{n}}-3\right)$$

For highly compressible materials, an additional term, f(J), is added for volumetric changes during compressive loading.

(2) $$W=\overset{\;}{\underset{n}{\sum }}\frac{{\tilde{\mu }}_{n}}{\alpha_{n}}\left(\lambda_{1}^{\alpha_{n}}{+\lambda }_{2}^{\alpha_{n}}{+\lambda }_{3}^{\alpha_{n}}-3+f\left(J\right)\right)$$

where J is the determinant of the deformation gradient and represents volumetric changes during compression. For incompressible materials, this parameter is equal to 1.

(3) $$J=\frac{\rho_{0}}{\rho }{=\lambda }_{1}\lambda_{2}\lambda_{3}$$

(4) $$f\left(J\right)=\frac{1}{\beta_{n}}\left(J_{\mathrm{el}}^{{-\alpha }_{n}\beta_{n}}-1\right)$$

Storakers proposed this formulation of the volumetric parameter for highly compressive porous lattice structures, where the initial shear and bulk modulus of the material are given by Equations (5) and (6), respectively.

(5) $$\mu_{0}=\underset{n}{\sum }\mu_{n}$$

(6) $$k_{0}=\underset{n}{\sum }2\left(\frac{1}{3}{+\beta }_{n}\right)\mu_{n}$$

Stable conditions, which are outlined by Storakers and others, for the material are enforced by requiring parameter relationships seen in Equations (7) and (8) [11,15].

(7) $$\mu_{n}\alpha_{n}>0$$

(8) $$\beta_{n}>-1/3\;$$

This ensures stability of the hyperelastic material model, which is also required during the mathematical fitting process. When this hyperelastic material is strictly undergoing uniaxial compression, the stress can be defined as a function of the principle stretch relation, which is shown in Equation (9).

(9) $$\sigma_{\mathrm{ii}}=\frac{2}{\lambda_{1}}\underset{n}{\sum }\frac{\mu_{n}}{\alpha_{n}}\left(\lambda_{1}^{\alpha_{n}}{-J}^{{-\alpha }_{n}\beta_{n}}\right)$$

**Figure 1:**
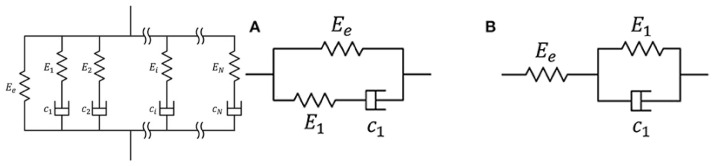
(**A**) Generalized Maxwell material model (**left**) with SLS or Zener model represented in Maxwell (**center**) and (**B**) Kelvin–Voigt (**right**) forms (Lin) [16].

The PVC gel is naturally a two-phase material containing a solid polymerized lattice with liquid plasticizer. Due to this two-phase nature, the material exhibits both viscosity and elasticity. This viscoelastic behavior is modeled through a generalized Maxwell viscoelastic material model. This model contains a spring with any number of dashpot–springs in parallel, as shown in Figure 1. By selecting a single dashpot–spring in parallel with the original spring, the material is known as a standard linear solid (SLS) or Zener material model. This SLS model can be represented in the Maxwell or Kelvin–Voigt forms, also shown in Figure 1.

The mathematical derivation of this model stems from the generalized Maxwell viscoelastic material model, which has a number of decaying exponential terms, but the SLS viscoelastic stress relaxation is shown in Equation (10).

(10) $$\sigma (t)=\epsilon_{0}\left(E_{E}{+E}_{1}e^{-\frac{t}{\tau_{1}}}\right)\;$$

This describes the exponentially decaying load that is seen in experimental compressive strain applications, which is drawn from the single dashpot–spring, also known as the Maxwell arm, of the Maxwell representation of the SLS viscoelastic material model. The response is also fit with a decaying exponential function, which is more phenomenalistic but assumes that the mechanoelectrical transduction properties are directly related to the viscoelastic stress relaxation. This could potentially lead to investigation of diffusion characteristics of liquid plasticizer to relate both the mechanical and electrical properties of these gel sensors.

The transport properties of these gel sensors are modeled by the Langmuir adsorption isotherm. This model has some simplified physics-based assumptions but is used to model a wide range of adsorptive phenomena. This model assumes that there is a single layer of liquid adsorbate into a solid interface, with no molecular interactions between the adsorptive species. The model assumes a first-order process for both adsorption and desorption to form the interfacial layer, and the surface morphology is not accounted for. Despite these simplified physics, this model remains one of the most prevalent adsorptive mathematical models and is used extensively in the explanation of a wide range of adsorptive phenomena. The Langmuir adsorption isotherm is given in Equation (11).

(11) $$\Theta =\frac{K_{S}c}{{1+K}_{S}c}\;$$

where the adsorptive equilibrium constant, $K_{S}$, is described by the first-order rate of adsorption and desorption, which are shown in Equations (12) and (13).

(12) $$N_{\mathrm{ads}}{=k}_{\mathrm{ads}}{\mathrm{cc}}_{\mathrm{sat}}\left(1-\Theta \right)$$

(13) $$N_{\mathrm{des}}{=k}_{\mathrm{des}}c_{\mathrm{sat}}\Theta$$

At equilibrium, these surface reaction rates are reduced to the Langmuir isotherm where the equilibrium constant, $K_{S}$, is equal to the adsorption rate constant, $k_{\mathrm{ads}}$, divided by the desorption equilibrium constant, $k_{\mathrm{des}}$. The transient response seen in experimentation also has an exponentially decaying-like overshoot attributed to dynamic effects of the gel. This exponentially decaying term, or dynamic term $J_{\mathrm{dyn}}$, is added to the rates of adsorption and desorption from the surface. This complete surface reaction rate, both transient and steady-state, is modeled in Equations (14) and (15).

(14) $$J_{\mathrm{ads}}{=k}_{\mathrm{ads}}\epsilon c_{\mathrm{sat}}\left(1-\Theta \right){+J}_{\mathrm{dyn}}$$

(15) $$J_{\mathrm{des}}{=k}_{\mathrm{des}}c_{\mathrm{sat}}{\Theta +J}_{\mathrm{dyn}}$$

where the dynamic term will exponentially decay, as seen in experimentation, and is described by Equation (16). The resulting steady-state adsorption rate will be dependent on the Langmuir adsorption isotherm.

(16) $$J_{\mathrm{dyn}}{=k}_{\mathrm{dyn}}\epsilon e^{-\frac{t}{\tau }}$$

This adsorptive phenomenon is used to describe the migration of polar plasticizer to the surface when undergoing compressive strain in the PVC gel material. This assumes that the applied strain causes an overall increase in volumetric concentration of plasticizer when the gel is compressed. The Storakers material model also assumes the high compressibility of the polymer lattice when undergoing compressive deformation. The adsorbed species to the interfacial layer is assumed to polarize due to uneven body forces at the surface of a polar fluid.

**Figure 2:**
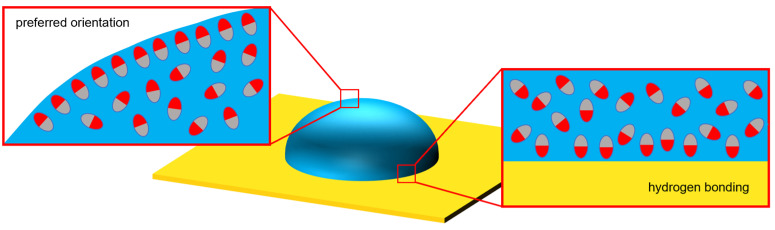
Illustration of interfacial polar molecule behavior and preferred orientation (**left**) and substrate effects such as hydrogen bonding on polar molecules (**right**).

This polarization can also be due to weak bonding to the substrate, which would be the copper electrode surface. These potential surface interactions are illustrated in Figure 2.

The electrical potential is calculated by the multiplying the fractional occupancy of adsorptive sites, from the Langmuir adsorption isotherm, by a proportionality constant $S_{E}$. This proportionality constant contains many physical properties such as dipolar strength of the plasticizer molecule, molecular packing parameter at the interfacial layer, and that the mechanoelectrical response is directly proportional to the fractional occupancy of the adsorptive sites in the interfacial layer. This describes the steady-state behavior of the mechanoelectrical transduction in the soft polymer gel materials. The transient response is also fit with a decaying exponential function, which is more phenomenalistic, but fits the data well. This may physically be due to electrostatic pressures, transient adsorptive phenomena, contact electrification, or other electrostatic effects.

The electric field created from this adsorptive-like phenomena results in some additional dipolar interactions in the material. Plasticizer is known to migrate under applied electric fields, so this field will cause some migration of interstitial plasticizer. This diffusion will also be classically described by the gradient of the concentration, as shown in Equation (17).

(17) $$J_{j}{=-D}_{j}\nabla c_{j}{-z}_{j}u_{m,j}{\mathrm{Fc}}_{j}\nabla V$$

This diffusion model contains two terms describing the migration under an electric field and concentration-dependent diffusion. The mobility, $u_{m,j}$, is given by the Nernst–Einstein relation which describes the mobility under an applied electric field as a function of the diffusion coefficient, $D_{j}$.

## 2. Materials and Methods

PVC gel samples were fabricated using dibutyl adipate (DBA) plasticizer at various weight ratios. The naming conventions used are shown in Table 2.

**Figure 3:**
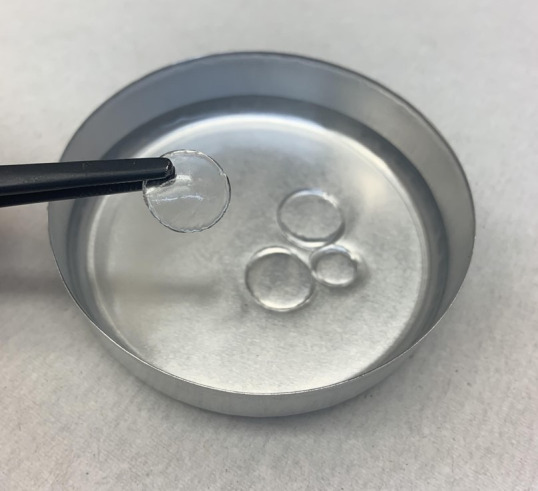
Fully cured PVC DBA P4 gel tray with punched sample to limit irregularities such as edge effects during the curing process.

The casting method was completed using tetrahydrofuran as the solution solvent. PVC and liquid plasticizer were mixed for 3 h prior to pouring into a casting dish. The casting dish was left undisturbed for 3 days for complete THF evaporation. An image of a PVC gel sample and casting dish is shown in Figure 3.

**Figure 4:**
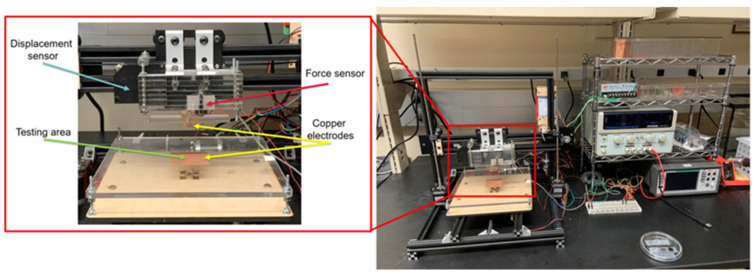
A detailed view of the force application mechanism with associated equipment for mechanoelectrical testing (**right**) including magnified region of soft polymeric sensing region (**left**).

Experimental testing was completed on a modified 3D printer setup to allow for precise strain applications to the gel samples with a 10 µm resolution. This force application mechanism is displayed in Figure 4.

An integrated load cell (Transducer Technologies GS0-250) and Keithley DAQ6510 data acquisition device collect both force and response data from the PVC gel samples while the plunger is incrementally lowered onto the gel sample, providing compressive strain application. The fabrication method for these PVC gel samples and the experimental setup are similar to the methods used in prior experiments investigating PVC gel sensor characteristics [9]. Environmental conditions were not investigated and may have some dependence on mechanoelectrical transduction within PVC gel sensors.

**Figure 5:**
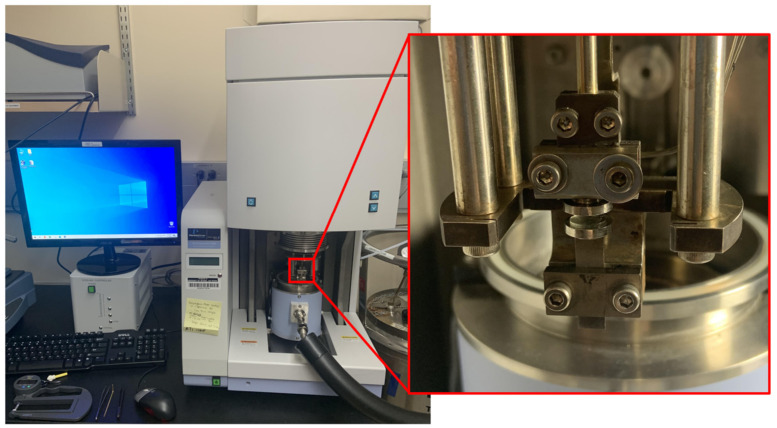
Dynamic mechanical analyzer (**left**) with magnified testing region of PVC gel sample (**right**) to determine stress–strain relationship under compressive loading applications.

A dynamic mechanical analyzer (Perkin Elmer Pyris Diamond DMA) was also used to construct a stress–strain relationship for each of the gel samples. The effects of the differing types of plasticizers on the material modulus might differ across tested P4 samples. This was performed to isolate the effect of plasticizer type on the material properties of gel samples. Additionally, the weight ratio of these samples may be equal across all tested P4 samples, but the molecular weight of each of these plasticizers differs. This potentially relates to different amounts of free interstitial plasticizer and volumetric differences in plasticizer migration. An image of this setup is given in Figure 5.

**Figure 6:**
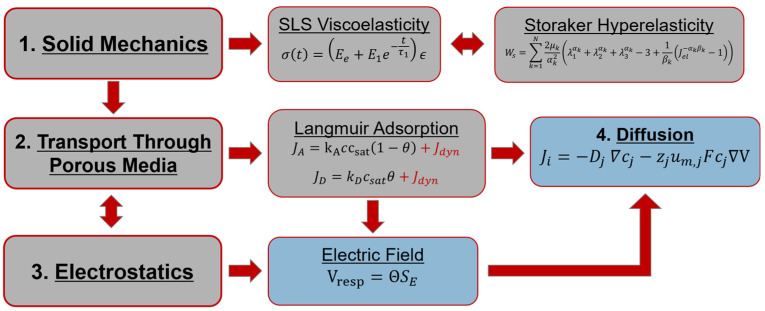
Flow chart of multiphysics model including the three major physics modules included in this study with plasticizer concentration gradient and electric potential response (blue).

The flow chart illustrated in Figure 6 describes the overview of the mathematical model with outputs including the electric field from the electrostatics module and the concentration gradient of plasticizer content calculated from the diffusion equation in the transport physics module. The built-in COMSOL physics modules are used to describe modeled physics which includes coupling between the solid mechanics, transport through porous media, and electrostatics governing equations.

**Figure 7:**
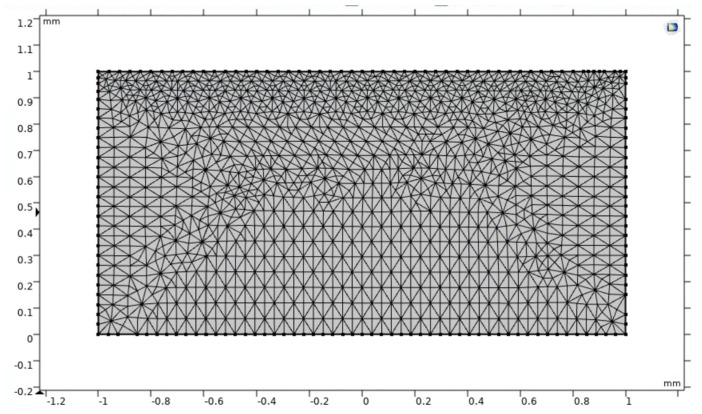
COMSOL model initial mesh including increased density near the surface due to modeled adsorptive surface phenomena.

A fixed boundary condition is placed on the lower boundary of the modeled gel sample. A ramped prescribed displacement is placed on the upper boundary, simulating the dynamic and static portions of the experimental strain application. This simulated ramped prescribed displacement is held constant once it reaches the desired level of compressive strain, which essentially models both the transient and steady-state compressive loading application observed in experimentation. The initial concentration of plasticizer in the gel sample is assumed to be homogenous prior to the strain application. The mesh is a free triangular mesh with an increased density near the surface due to the surface phenomena associated with the modeled physics. This mesh can be observed in Figure 7. A mesh refinement is also completed at each time step to ensure adequate nodal mapping.

## 3. Results

### 3.1. Solid Mechanics Study

**Figure 8:**
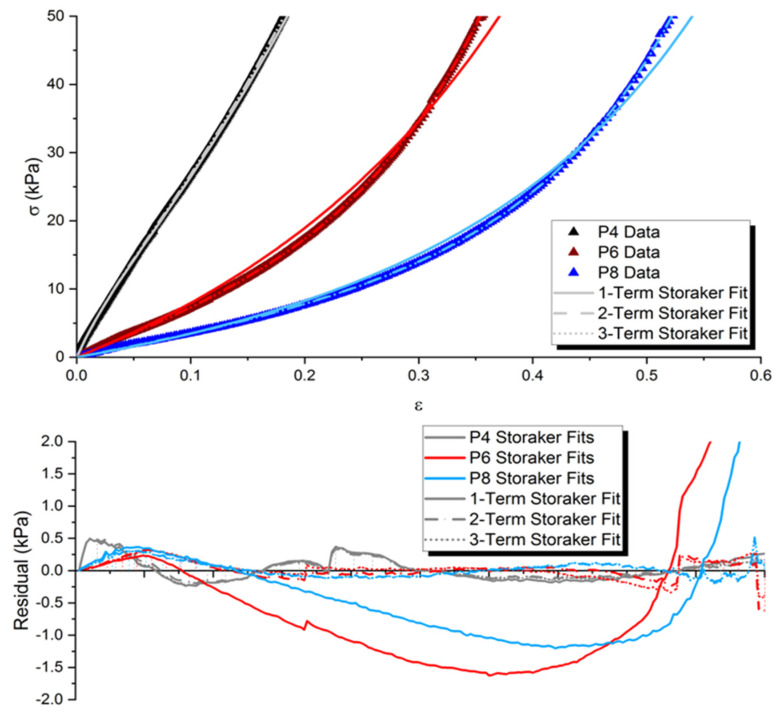
DMA results for P4, P5, and P6 samples with one-, two-, and three-term Storakers hyperelastic material model fits (**top**), with residuals plot (**bottom**) showing good agreement among experimental results for this compressive hyperelastic material model.

One-, two-, and three-term Storakers material models were fit to P4, P6, and P8 data obtained from the DMA testing using a nonlinear least-square fitting method. Error analysis was completed using the residuals for each mathematical fit. These data are shown in Figure 8 and Table 3. Table 3 shows both the average of the absolute value of the error as a percentage across all data points and the maximum residual in terms of kPa.

The one-term compressible hyperelastic Storakers material model fits the data fairly well, specifically in the P4 sample with lower plasticizer content. The stress–strain relation of the PVC P4 sample is much more linear when compared to higher levels of plasticizer, indicating a lower level of plasticization of the polymeric lattice. This one-term model begins to fail at increasing levels of nonlinearity and is observed in the large residuals for P6 and P8 fits. The two-term model seems to fit data across all samples very well with a maximum error of 0.646 kPa, which is observed in the P6 dataset. This error is also a function of the real data obtained from the DMA, which inherently contains errors and uncertainties and is apparent in the irregular craggy shape of the residuals plot. However, this two-term model well describes all plasticizer ratios used in this test from the pseudolinear P4 relationship to the highly nonlinear P8 samples denoted in bold in Table 3. The three-term fit seems to not add any additional accuracy of the model and provides an absolute maximum error of 0.513 kPa.

This marginal decrease in error is not necessary for the robust hyperelastic material model. A two-term Storakers hyperelastic material model was chosen to describe the hyperelastic compressive stress–strain relationship in this soft polymeric gel mechanoelectrical transduction model.

**Figure 9:**
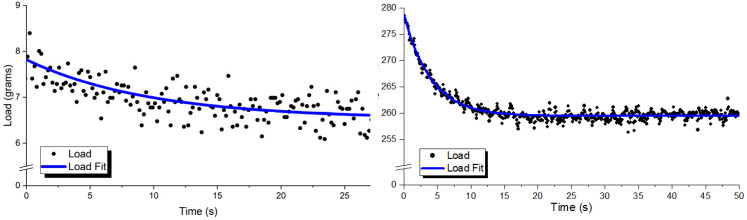
Viscoelastic stress relaxation observed in PVC gel samples when undergoing stepped compressive strain input (black) and SLS model fit (blue) for low (**left**) and high (**right**) compressive loading applications.

The viscoelastic properties are also modeled using the Maxwell form of the SLS material model. This is due to the exponentially decaying viscoelastic stress relaxation observed in experimentation, as shown in Figure 9. In the upper portion of this figure, the SLS model is fit to viscoelastic stress relaxation in the low-force region. This region typically shows less viscous behavior and has a smaller magnitude of the Maxwell arm relating to the overshoot in stress from a stepped strain input. The lower portion of this figure shows the viscoelastic stress relaxation in the higher force region where more viscous effects are observed, as seen by the larger stress decay.

All regions in the experimental region, including these two, are well described by the SLS viscoelastic stress relaxation. This Maxwell arm describing the decaying portion of the viscoelastic stress relaxation varies over strain application, as seen in the figure, but also varies over plasticizer type and weight ratio as well. However, if the material properties of the gel sensor are known, then this viscoelastic response is predictable. This exponentially decaying stress relaxation is observed in all experimentally tested polymeric gel sensors in the tested region and can be described using this mathematical function.

### 3.2. Transport and Migratory Effects

The adsorption phenomenon is modeled as dynamic surface reactions within the COMSOL Multiphysics software. This surface reaction is based on the applied strain and also contains the phenomenalistic exponentially decaying dynamic response that occurs in experimentation. The strain-dependent surface reaction assumes that the applied strain directly correlates to a volumetric change in concentration due to high compressibility of the PVC lattice. This direct correlation is supported by the limited transverse effects imaged in the tested loading region. The transient response seen in experimentation also has an exponentially decaying-like overshoot attributed to dynamic effects of the gel. This complete surface reaction rate, both transient and steady-state, is modeled by the modified Langmuir adsorption and desorption rate equations described in Equations (13)–(16). These physics assume the PVC lattice to be electrostatically inert and serve strictly as a compressible porous medium for mobile liquid plasticizer undergoing adsorptive phenomena.

**Figure 10:**
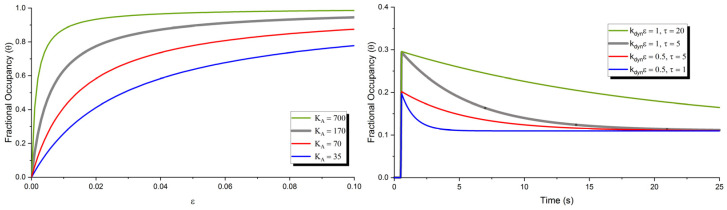
Variation of steady-state response parameter $K_{A}$ (**left**) in adsorption-based mathematical model and transient response parameters $k_{\mathrm{dyn}}$ and τ (**right**) displaying effects on fractional occupancy of adsorption sites Θ.

This describes both the transient and steady-state fractional occupancy of adsorptive sites at the interfacial layer where the maximum saturated surface concentration is given by $c_{\mathrm{sat}}$. The phenomenalistic mathematical model for the transient response, $J_{\mathrm{dyn}}$, will decay to leave the Langmuir adsorption isotherm to describe the steady-state mechanoelectrical response of these gels dependent on applied strain. Again, it is assumed that in this experimental region, the applied compressive strain directly correlates to a volumetric change in plasticizer concentration. This dynamic response effect that is modeled by a phenomenalistic exponential decay is potentially a function of plasticizer type and weight ratio and applied strain and associated rate of application. An extensive study is required to fully characterize this transient response. Some variation in fit parameters $K_{A}$, $k_{\mathrm{dyn}}$, and τ is shown in Figure 10 for various steady-state and dynamic responses observed in experimentation. The $K_{A}$ parameter will affect the rate of saturation of adsorption sites and the steady-state response saturation rate. The $k_{\mathrm{dyn}}$ and τ parameters will affect the initial overshoot and decay rate of the transient response. The electric potential and field created from this migration of polar plasticizer at the interfacial layer is described by the proportionality constant, $S_{E}$. The electrostatics module is used to describe the temporal evolution of the electric field. This assumes the complete polarization of adsorbed dipolar plasticizer at the interface. This electric field calculation is then used in the transport module to model additional diffusive effects of the bulk.

### 3.3. Model Results with Experimental Validation

**Figure 11:**
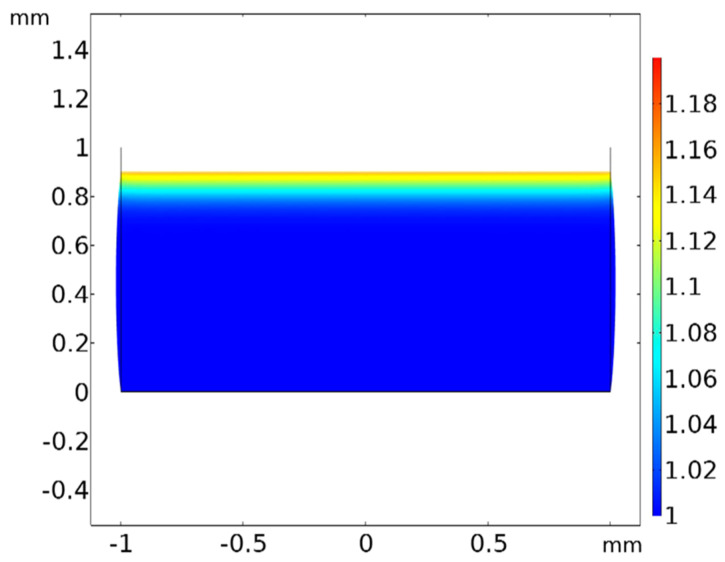
Steady-state relative concentration plot of compressed PVC gel sample with 0.1 compressive strain displaying higher plasticizer concentrations in a layer approximately 100 μm near the interfacial layer.

The modeled relative plasticizer concentration through the thickness of a compressed sample is shown in Figure 11. This theorized concentration gradient is due to adsorbed interfacial layer coupled with the mobility of free plasticizer. This gradient is a direct result of the electric field induced by the adsorbate.

**Figure 12:**
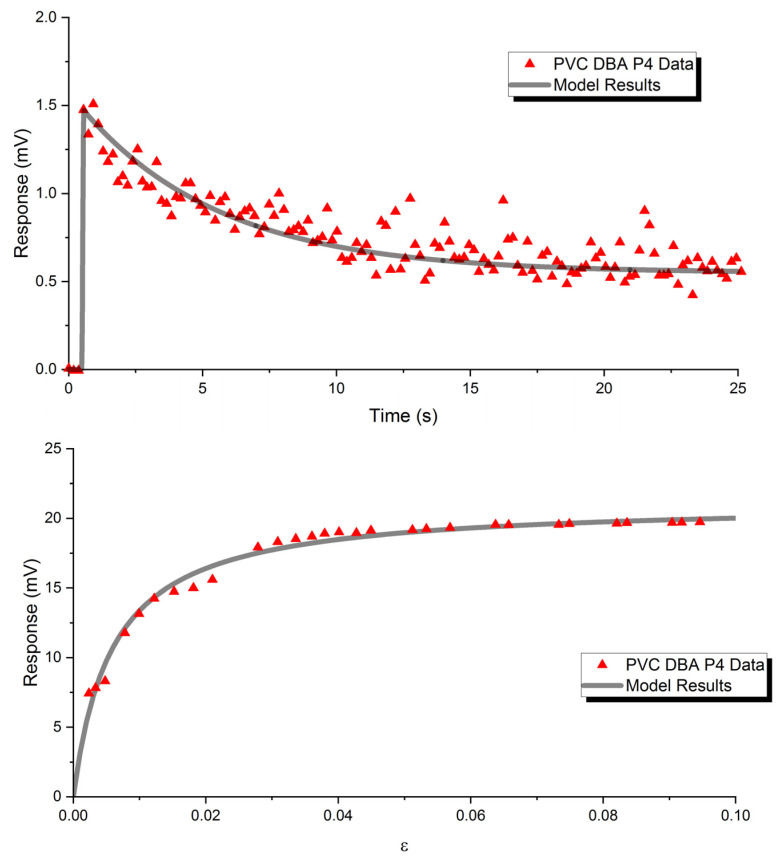
Obtained experimental data and mathematical model results for transient (**top**) steady-state (**bottom**) response characteristics showing ability of the model to reflect experimental results of soft polymer gel sensors.

This initial mathematical model may also be useful in predicting behavior of soft polymer gels with known qualities. The transient and steady-state mechanoelectrical response seen in experimentation due to a step input in strain and the resulting mathematical model can be seen in Figure 12. Specific adsorptive parameter values were obtained using parametric sweeps of transient and steady-state adsorptive variables. A coarse sweep of transient and steady-state adsorptive coefficients can be observed in Figure 10.

The mathematical model does show accuracy for both transient and steady-state results; however, the transient response is currently modeled through a phenomenalistic approach. This transient behavior could deviate from this phenomenalistic approach for the dynamic response. For instance, in experimentation it is shown that this initial overshoot relates to the plasticizer content of the gel sample. This complex dynamic response is also a function of strain rate application and total strain applied. A systematic study to characterize the complex dynamic response seen in the gels could prove to yield a mathematical model rooted in underlying physical mechanisms and/or provide adsorptive coefficient values for transient behaviors to strengthen the current model. However, the steady-state response seems to be modeled very well over many types of plasticizers and multiple polymer lattice structures using the Langmuir adsorption modeling approach. The apex parameters for prediction of mechanoelectrical performance of soft polymeric gel sensors seem to be the cross-sectional area of the gel sensor, plasticizer type and weight ratio, polymer lattice material, dipolar strength of plasticizer, plasticizer molecular packing parameter, and plasticizer electrode wettability. With this knowledge, basic material characterization can be completed on an arbitrary plasticizer and polymer structure for predictive performance of soft polymer gel sensors.

## 4. Discussion

This mathematical model provides a foundation for multiphysics modeling of soft polymeric gel sensors. This model begins with the solid mechanics physics of the soft polymeric gel sensor. The viscoelastic properties observed in experimentation are modeled through the use of the SLS model. This model accurately predicts and has been fit to many experimental datasets including variable plasticizer types and weight ratios, as well as variable polymeric lattice structures, to a high degree of accuracy. The hyperelasticity was experimentally investigated under uniaxial compression in the dynamic mechanical analyzer, and a highly compressive Storakers model was used to describe this nonlinear stress–strain relationship. Lower plasticizer content results in higher modulus and more linearity in the experimental region. Even a one-term fit modeled this stress–strain relationship through a range of plasticizer weight ratios; however, a two-term fit better modeled increasing plasticizer weight ratio samples exhibiting higher degrees of nonlinearities. This material model is extensive and models a wide range of soft polymeric gel sensors.

The transport of fluid media through a porous lattice has two major components, one being the adsorptive-like behavior and the second portion describing the bulk migration. The adsorptive-like behavior based on a Langmuir model defines the steady-state mechanoelectrical behavior of these materials very well. This does assume high compressibility and total polarization of adsorbed species but can be used to predict performance of soft polymeric gel sensors with known material properties. However, the underlying physics of the transient or dynamic response is currently unknown. This model can show the exponential decay observed in experimentation but has no physical basis and is a current weakness in the model. The bulk migration is modeled though the classical diffusion equation with terms relating to the concentration gradient and the migration due to the induced electric field. Imaging of this was attempted using three separate imaging techniques. Currently this bulk concentration migration is unknown, but is probable due to known electroactive phenomena. In addition, plasticizer migration can be observed during the SEM imaging technique using the in situ compression stage [9].

Currently, this model includes an extensive material model accurately modeling the transient and steady-state behaviors of these soft polymeric gels in uniaxial compression. The steady-state mechanoelectrical behavior can be predicted for arbitrary plasticizer and polymer lattice structures using the Langmuir adsorption isotherm with some known material properties. These are clearly the strengths of the current mathematical model. Some underlying studies will need to be completed to fully grasp the transient dynamic response. In addition, some investigation of bulk properties could enhance the known plasticizer concentration gradient. This model does, however, provide a foundation for multiphysics modeling of soft polymeric gel sensors.

## Figures and Tables

**Table 1:** Mathematical nomenclature.

Variable	Units	Description
ϵ	$\left[-\right]$	Strain on polymer gel sample
ρ	$\left[{\mathrm{kg}/m}^{3}\right]$	Density
Θ	$\left[-\right]$	Fractional occupancy of adsorptive sites
$V_{\mathrm{resp}}$	$\left[V\right]$	Voltage response
c	$\left[{\mathrm{mol}/m}^{3}\right]$	Concentration of bulk species
$K_{S}$	$\left[-\right]$	Equilibrium constant of surface layer
$S_{E}$	$\left[V\right]$	Proportionality constant
τ	$\left[s\right]$	Time constant
$c_{\mathrm{sat}}$	$\left[{\mathrm{mol}/m}^{2}\right]$	Saturated surface concentration
$k_{\mathrm{ads}}$	$\left[m^{3}/\mathrm{mol}\cdot s\right]$	Adsorption rate constant
$k_{\mathrm{des}}$	$\left[1/s\right]$	Desorption rate constant
$N_{\mathrm{ads}}$	$\left[{\mathrm{mol}/m}^{2}\cdot s\right]$	Rate of adsorption
$N_{\mathrm{des}}$	$\left[{\mathrm{mol}/m}^{2}\cdot s\right]$	Rate of desorption
$\lambda_{1,2,3}$	[−]	Principal stretches
$\mu_{n}$	$\left[{N/m}^{2}\right]$	Shear modulus
$\alpha_{n}$	$\left[-\right]$	Storakers model parameter
$\beta_{n}$	$\left[-\right]$	Storakers model parameter
J	$\left[-\right]$	Volumetric parameter
σ	$\left[{N/m}^{2}\right]$	Stress
$k_{n}$	$\left[{N/m}^{2}\right]$	Bulk modulus
W	$\left[{J/m}^{3}\right]$	Strain energy density function
$J_{\mathrm{ads}}$	$\left[{\mathrm{mol}/m}^{2}\cdot s\right]$	Modeled adsorption rate
$J_{\mathrm{des}}$	$\left[{\mathrm{mol}/m}^{2}\cdot s\right]$	Modeled desorption rate
$J_{\mathrm{dyn}}$	$\left[-\right]$	Modeled dynamic interfacial effect
$k_{\mathrm{dyn}}$	$\left[-\right]$	Dynamic interfacial rate constant
E	$\left[{N/m}^{2}\right]$	Compressive modulus
t	[s]	Time
$D_{j}$	${[m}^{2}/s]$	Diffusivity of species j
$c_{j}$	${[\mathrm{mol}/m}^{3}]$	Concentration of species j
$z_{j}$	[−]	Charge number of species j
$u_{m,j}$	${[m}^{2}/s\cdot V]$	Mobility of species j in medium m
F	[C/mol]	Faraday constant
V	[V]	Electric potential

**Table 2:** PVC gel composite weight ratios and naming convention.

Name	P4	P6	P8
PVC: plasticizer ratio	1:4	1:6	1:8

**Table 3:** Error analysis of Storakers hyperelastic material model.

Model Fit	P4 Error	P6 Error	P8 Error
One-term	3.04% (0.497 kPa)	6.96% (4.094 kPa)	7.58% (4.198 kPa)
**Two-term**	**3.07% (0.505 kPa)**	**1.83% (0.646 kPa)**	**3.02% (0.493 kPa)**
Three-term	3.02% (0.499 kPa)	1.71% (0.430 kPa)	3.02% (0.513 kPa)

## Data Availability

The data that support the findings of this study are available upon reasonable request from the authors.
